# Atrial Fibrillation Characteristics in Patients on Haemodialysis vs. Peritoneal Dialysis

**DOI:** 10.1038/s41598-018-21229-9

**Published:** 2018-02-14

**Authors:** Ran Abuhasira, Yuval Mizrakli, Avi Shimony, Victor Novack, Alla Shnaider, Yosef S. Haviv

**Affiliations:** 1 The Joyce and Irving Goldman Medical School, Faculty of Health Sciences, Ben-Gurion University, Beer-Sheva, Israel; 2 Clinical Research Centre, Soroka University Medical Centre, Beer-Sheva, Israel; 3 Department of Cardiology, Soroka University Medical Centre, Beer-Sheva, Israel; 4 Department of Nephrology, Soroka University Medical Centre, Beer-Sheva, Israel; 5 Faculty of Health Sciences, Ben-Gurion University, Beer-Sheva, Israel

## Abstract

Atrial fibrillation (AF) is highly prevalent in dialysis patients, however whether its impact differs between patients on haemodialysis (HD) vs. peritoneal dialysis (PD) is unknown. We aimed to compare the association of AF and clinical outcomes in different dialysis modalities. We performed a population based retrospective cohort study, including adult patients who initiated dialysis between the years 2002 and 2015. Clinical, echocardiographic and laboratory data were reviewed and correlated with outcomes in HD vs. PD. During the study period, 1,130 patients began dialysis. Of the 997 patients without AF before dialysis initiation, 17% developed new-onset AF after the initiation of dialysis (17.3% of HD vs. 13.7% of PD patients, p = 0.27). Using multivariate analysis, only enlarged left atrium at dialysis initiation (hazard ratio (HR) 2.82, CI95% 2.00–3.99) and age (HR 1.04, CI95% 1.03–1.06) were significantly associated with AF. Dialysis modality was not a significant predictor of AF in either univariate or multivariate analysis. In conclusion, our study demonstrated that AF is common in dialysis patients irrespective of modality. In our cohort, the risk factors associated with AF were older age and enlarged left atrium. AF was associated with increased rates of heart failure and mortality, but not stroke.

## Introduction

Atrial fibrillation (AF) is a common arrhythmia in the general population and in end stage renal disease (ESRD) patients. The prevalence of AF in the general population ranges from 2.3–3.4% and is expected to double by 2050^1^. The prevalence of AF in haemodialysis (HD) patients ranges from 3.8–27%^2^. The incidence of AF in ESRD patients is substantially higher than in the general population^3^, and was reported to be higher in HD patients compared to peritoneal dialysis (PD) patients^4,5^. The incidence varies from 0.5 per 100 person-years to 14.8 per 100 person-years^3^. According to previous studies, the risk factors for AF in ESRD patients were primarily age, followed by hypertension, heart failure, coronary artery disease, peripheral vascular disease and chronic obstructive pulmonary disease^3,6^. The risk for stroke in HD patients is 5 to 10 times higher than the risk for subjects with a normal kidney function^7^, but the association with AF is inconclusive. This contrasts with AF in the general population, where AF is a strong risk factor for stroke^8^. Importantly, AF is associated with a higher mortality risk in dialysis patients^9^.

Most studies relating to AF in ESRD patients have either focused on HD patients or evaluated ESRD patients as one group. Abbott *et al*.^4^ assessed the effect of the different dialysis modalities on the development of AF and associated outcomes in hospitalized dialysis patients, but comparison of AF in HD vs. PD has not been previously reported in outpatient dialysis patients. We conducted a long-term population-based cohort study to compare the risk factors for developing AF and the associations of AF with heart failure, stroke and mortality in chronic HD and PD patients.

## Materials and Methods

### Study design

We performed a population based retrospective cohort study. The Nephrology Department in our University Medical Centre oversees all dialysis services provided to the entire population of ~700,000 people living in Southern Israel (Negev district). We included in the study from January 1, 2002 to December 31, 2015, all the members of “Clalit”, health maintenance organization (HMO) that owns the hospital, who initiated dialysis therapy during the study period and were 18 years of age or older. Patients that are not members of “Clalit” were excluded due to limited access to data regarding their diagnoses, medications and laboratory results. Patients were excluded if their dialysis vintage or total follow-up time were shorter than 90 days.

### Study population, clinical definitions and data sources

Following a previous report by Liem Y *et al*.^10^, we used a univariate analysis to define the type of dialysis modality on day 90 after the initiation of dialysis treatment (compatible with an intention-to-treat analysis). The exclusion of the first three months of dialysis was required to overcome confounding by acute kidney injury, early modality switch and many events of death occurring shortly after dialysis initiation. Patients were followed up until renal transplantation, death, or 31 December 2015.

We identified patients with evidence of ESRD and dialysis treatment based on International Classification of Diseases, 9^th^ revision (ICD-9) codes. Data on ICD-9 diagnoses in the hospital and diagnoses in community clinics, demographic data, laboratory tests results, echocardiography test results and medication purchases throughout the entire study period were captured from the HMO computerized database. Dialysis vintage was defined by the time difference between the initiation of a specific modality to the end of follow-up date or modality switch. HD patients were identified by ICD diagnoses, hospital billing records for at least 90 days and internal records of the Nephrology Department. PD patients were identified by ICD diagnoses of PD or insertion of cutaneoperitoneal fistula with a diagnosis of kidney disease and internal records of the Nephrology Department. The hospital’s treatment policy with HD or PD remained consistent throughout the study period. Warfarin usage was defined by purchase of the drug during the study period. Data on warfarin use are presented only for the HD group since there were very few users of warfarin in the PD group.

The outcomes were all-cause mortality, new-onset AF, major bleeding event and cerebrovascular event. The outcomes were defined by diagnosis codes signed exclusively after the date of dialysis initiation, i.e. events that occurred before the initiation of dialysis were not considered as outcomes.

A major bleeding event was defined by any of the following: 1) hospital diagnosis of any bleeding with a haemoglobin drop of at least 3 g/dL; 2) hospital diagnosis of any bleeding with a diagnosis of blood product transfusion; 3) hospital diagnosis of intra-cranial or intra-ocular haemorrhage^11^. Cerebrovascular events were defined as either ischemic stroke or transient ischemic attack (TIA). Diagnosis of atrial fibrillation was defined using the ICD-9 codes 427.3 and 427.31 given at the hospital. Of note, the ICD-9 codes do not differentiate between different types of atrial fibrillation.

We assessed the echocardiography tests done within one year of initiation of dialysis (available for 70% of the cohort). We included data on left ventricle systolic function, mitral regurgitation severity and left atrium size. Enlarged left atrium was defined according to a position paper endorsed by the European Society of Cardiology: parasternal long-axis view diameter larger than 41 mm or apical view major length larger than 57 mm^12^. We chose the old, yet validated linear parameters for left atrial enlargement as most of the data lacked data concerning left atrium volume.

### Statistical analysis

When appropriate, univariate comparisons were made using χ^2^-test or Fisher’s exact test for categorical variables, and using Student’s t-test or Mann–Whitney test for quantitative variables.

Patient survival times were compared between the dialysis modalities from the first dialysis date. Patients were left censored for the first 90 days and right censored at renal transplantation date if this event occurred during the follow-up time. The end of follow-up time was either date of death, 31 December 2015, or date of renal transplantation. Time to death and all other outcomes were presented by Nelson-Aalen cumulative hazard function and compared between subgroups using the log-rank test for univariate analysis.

For multivariate analysis, we used cause-specific Cox proportional hazards regression model with time dependent covariates. Variables were entered via a stepwise forward selection approach. The models included clinically and statistically significant (p < 0.1) variables. Clinically significant variables were set a priori and included the following: age, sex, dialysis modality, left atrium size, systolic function and evidence for moderate or severe mitral regurgitation. Additional variables included in the model: hypertension, chronic obstructive pulmonary disease, diabetes mellitus, heart failure, peripheral vascular disease and a history of myocardial infarction. Due to the potential changes in the health status of the patients over the study span, all the variables in the model were analysed in a time-varying fashion. We have constructed serial annual cohorts of the patients to determine the status of all the variables each year. Dialysis modality was defined for each year of the study as a categorical variable by the most recent dialysis diagnosis at each year. Systolic function was defined by the last echocardiography each year; in case there was not a new exam, the result of the previous year was used. Age was defined as a continuous variable. All other variables were defined as categorical and did not change in time after the first date of diagnosis (for example, once a diagnosis of hypertension was given, it could not be changed). Patients with a diagnosis of AF preceding dialysis initiation were excluded from the analysis of new-onset AF.

A p-value of 0.05 or less (two-sided) was considered statistically significant. IBM SPSS software, version 24.0, and Stata software, version 12, were used for statistical analysis.

### Ethics

The study was approved by the “Soroka University Medical Centre” institutional review board (IRB) Committee. All clinical investigations were conducted according to the principles expressed in the Declaration of Helsinki. The IRB approval exempted the study from informed consent due to the retrospective data collection nature maintaining subject confidentiality. Patient records were anonymised and de-identified prior to analysis.

### Data availability

The data used in the analysis of this study are not publicly available due to the requirements of the IRB Committee, but are available from the corresponding author upon request.

## Results

### Characteristics of the cohort

**Figure 1:**
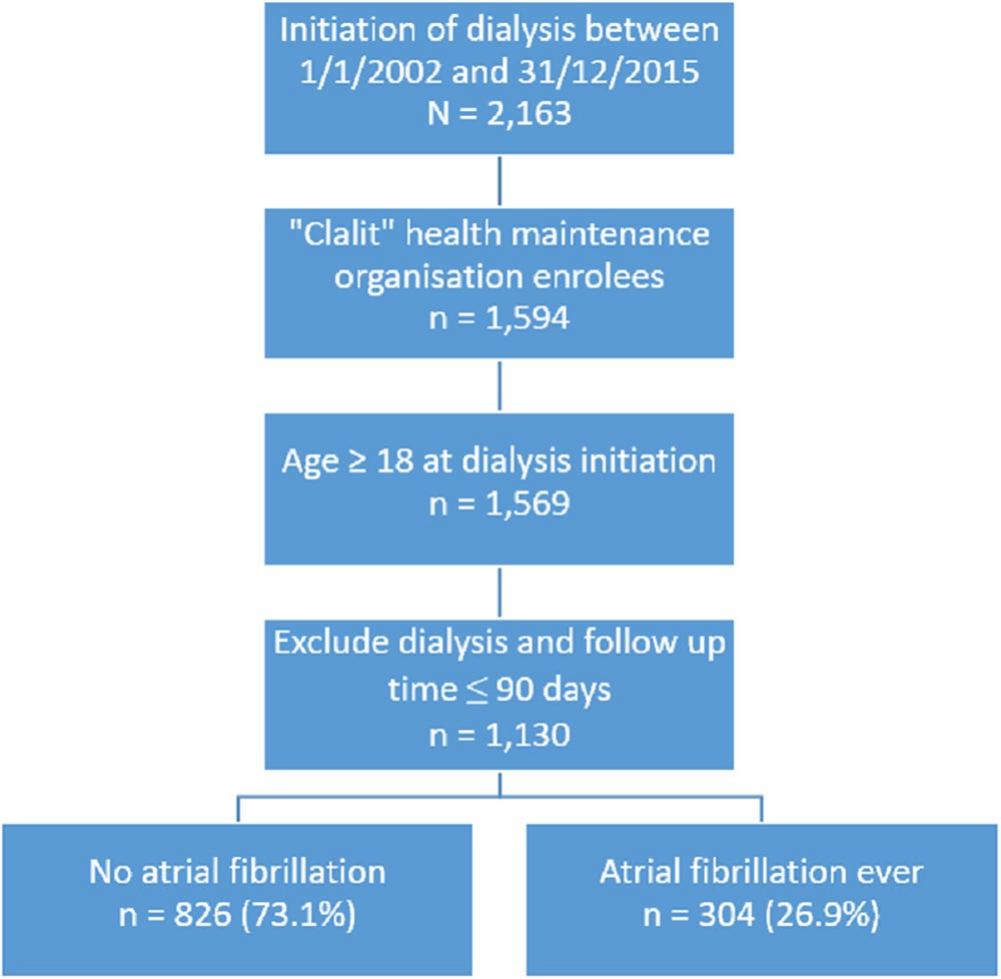
Flow chart for selection of study population.

Following the exclusion criteria depicted in the Methods section, we identified 1,130 patients undergoing dialysis during the study period, of whom 26.9% were diagnosed with AF before or after dialysis initiation (Fig. 1). We stratified the entire dialysis patient cohort by either ever receiving a diagnosis of AF or not, and evaluated the baseline characteristics at initiation of dialysis (Table 1). The patients with AF were significantly older (mean age 61.6 ± 15.3 in the non-AF group and 70 ± 10.6 in the AF group, p < 0.001) and suffered from more cardiovascular comorbidities than the patients without AF at baseline.

**Table 1:** Baseline characteristics by atrial fibrillation at dialysis initiation.

Group	All (N = 1,130)	No AF (n = 826)	AF Ever (n = 304)	P-value
Age (years)	63.8 ± 14.7	61.6 ± 15.3	70 ± 10.6	**<0.001**
Male (n, %)	661 (58.5%)	479 (58.0%)	182 (59.9%)	0.57
Haemodialysis (n, %)	1021 (90.4%)	738 (89.3%)	283 (93.1%)	0.06
Peritoneal dialysis (n, %)	109 (9.6%)	88 (10.7%)	21 (6.9%)	0.06
AF prior to dialysis (n, %)	133 (11.8%)	—	133 (43.8%)	—
Diabetes mellitus (n, %)	731 (64.7%)	505 (61.1%)	226 (74.3%)	**<0.001**
Heart failure (n, %)	275 (24.3%)	151 (18.3%)	124 (40.8%)	**<0.001**
Hypertension (n, %)	884 (78.2%)	629 (76.2%)	255 (83.9%)	**0.01**
Coronary artery disease (n, %)	441 (39.0%)	276 (33.4%)	165 (54.3%)	**<0.001**
Myocardial infarction (n, %)	230 (20.4%)	148 (17.9%)	82 (27.0%)	**0.001**
Pulmonary hypertension (n, %)	51 (4.5%)	21 (2.5%)	30 (9.9%)	**<0.001**
Dyslipidaemia (n, %)	901 (79.7%)	637 (77.1%)	264 (86.8%)	**<0.001**
Peripheral vascular disease (n, %)	133 (11.8%)	87 (10.5%)	46 (15.1%)	**0.03**
Intracranial haemorrhage (n, %)	20 (1.8%)	14 (1.7%)	6 (2.0%)	0.75
Cerebrovascular event (n, %)	119 (10.5%)	73 (8.8%)	46 (15.1%)	**0.002**
CHA_2_DS_2_-VASc score (median, interquartile range)	3 (2,5)	3 (2,4)	4 (3,5)	**<0.001**
Chronic obstructive pulmonary disease (n, %)	47 (4.2%)	29 (3.5%)	18 (5.9%)	0.07
Smoking (ever, n, %)	465 (41.2%)	347 (42.0%)	118 (38.8%)	0.33
Hyperthyroidism (n, %)	25 (2.2%)	20 (2.4%)	5 (1.6%)	0.43
Albumin (g/dl, average)	3.4 ± 0.6	3.5 ± 0.6	3.3 ± 0.6	**0.004**
Haemoglobin (g/dl, average)	10.3 ± 1.7	10.3 ± 1.7	10.3 ± 1.6	0.88
Mean follow-up time (months)	39.7 ± 32.3	40.1 ± 33	38.5 ± 30.5	0.46
Transplant after dialysis (n, %)	89 (7.9%)	78 (9.4%)	11 (3.6%)	**0.001**

Groups were defined by the diagnosis of atrial fibrillation ever, before or after dialysis initiation. All the comorbidities are prior to the initiation of dialysis. Albumin and haemoglobin results are within 30 days of dialysis initiation. AF – atrial fibrillation.

Univariate analysis showed that AF patients had significantly larger left atrium, worse systolic function and a higher rate of moderate or severe mitral regurgitation (Table 2).

**Table 2:** Echocardiography values by atrial fibrillation, before or after dialysis initiation.

Group	All (N = 1,130)	No AF (n = 826)	AF Ever (n = 304)	P-value
Enlarged left atrium* (n, %)	323 (28.6%)	171 (20.7%)	152 (50.0%)	<0.001
Systolic function (n, %)^*,†^ EF > 40% EF 30–40% EF < 30%	1012 (89.6%) 36 (3.2%) 82 (7.3%)	755 (91.4%) 25 (3.0%) 46 (5.6%)	257 (84.5%) 11 (3.6%) 36 (11.8%)	0.001
Mitral regurgitation – Moderate or severe (ever) (n, %)	165 (14.6%)	89 (10.8%)	76 (25.0%)	<0.001

*AF – atrial fibrillation*, EF – ejection fraction.

^*^Value most proximate to dialysis initiation within 1 year.

^†^No Echo values – equals normal.

Stratification of the cohort by dialysis modality, according to intention-to-treat analysis at day 90, showed that 28.3% of the HD group and 19.3% of the PD group ever received a diagnosis of AF. Comparison of the echocardiographic data between the HD and PD groups showed no significant difference between the groups at dialysis initiation (Table 3).

**Table 3:** Echocardiography values by dialysis modality.

Group	All (N = 1,130)	HD (n = 1,021)	PD (n = 109)	P-value
Enlarged left atrium (n, %)*	323 (28.6%)	292 (28.6%)	31 (28.4%)	0.97
Systolic function (n, %)*^,†^ EF > 40% EF 30–40% EF < 30%	1,012 (89.6%) 36 (3.2%) 82 (7.3%)	913 (89.4%) 36 (3.5%) 72 (7.1%)	99 (90.8%) 0 (0.0%) 10 (9.2%)	0.77
Mitral regurgitation–Moderate or severe (ever) (n, %)	165 (14.6%)	146 (14.3%)	19 (17.4%)	0.38

Groups were defined by the modality of dialysis at day 90 after dialysis initiation. HD – Haemodialysis, PD–Peritoneal dialysis, EF – ejection fraction.

^*^Value most proximate to dialysis initiation within 1 year.

^†^No Echo values – equals normal.

### Outcomes associated with atrial fibrillation

**Figure 2:**
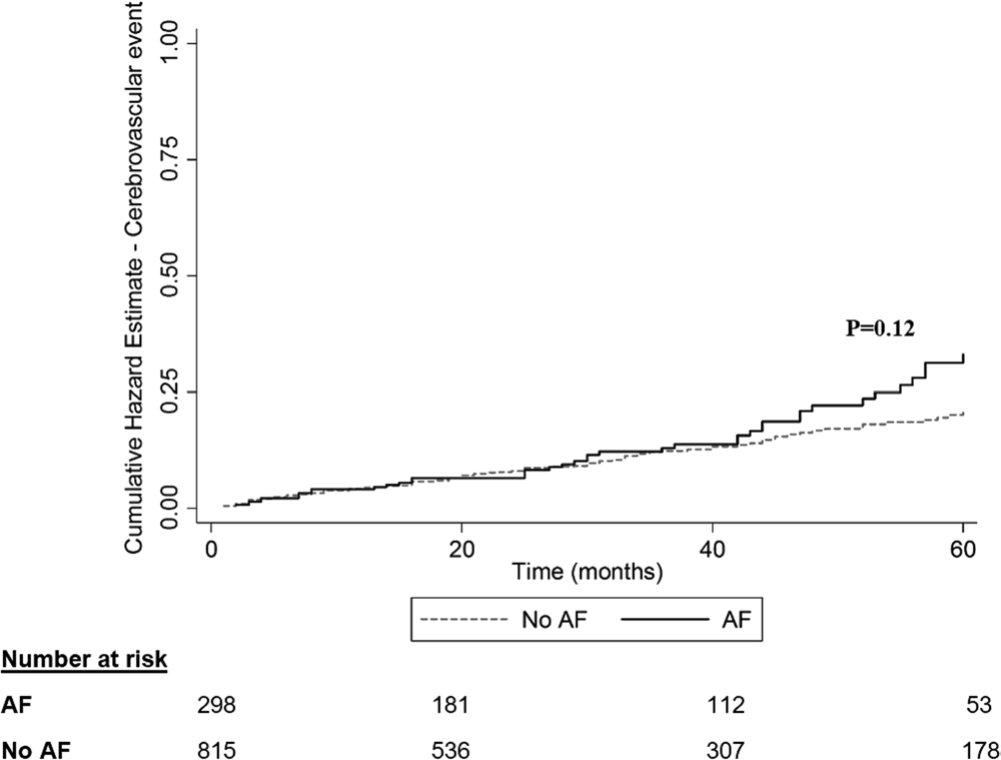
Cerebrovascular event (ischemic stroke and transient ischemic attack, TIA) Nelson-Aalen cumulative hazard estimate grouped by atrial fibrillation ever. Includes only diagnoses of stroke and TIA given after dialysis initiation. Diagnosis of atrial fibrillation was given before or after dialysis initiation. AF – atrial fibrillation.

**Figure 3:**
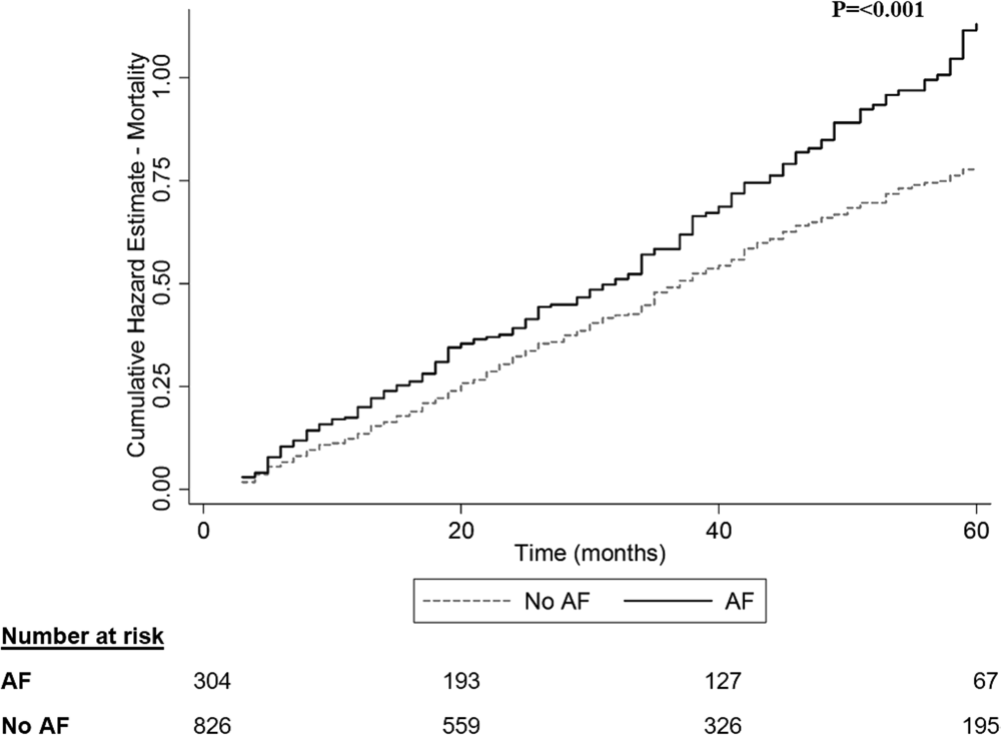
All-cause mortality Nelson-Aalen cumulative hazard estimate grouped by atrial fibrillation ever.Diagnosis of atrial fibrillation was given before or after dialysis initiation. AF – atrial fibrillation.

The rates of cerebrovascular events, ischemic stroke and TIAs, were similar when comparing dialysis patients with AF and without (16.4% in the AF group, 13.4% in the non-AF group, p = 0.12, Fig. 2). Notwithstanding the similar rates of cerebrovascular events, AF was associated with a higher mortality rate (73.7% in the AF group, 55.1% in the non-AF group, p < 0.001, Fig. 3), with differences in mortality starting early after dialysis onset.

### Differences between dialysis modalities

**Figure 4:**
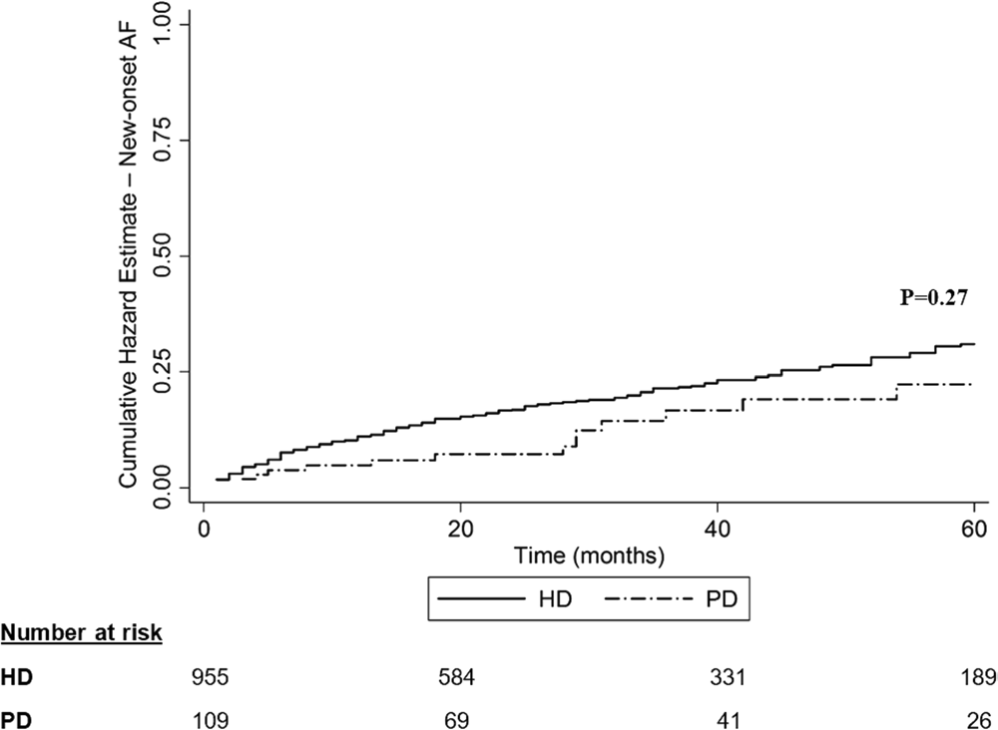
New-onset atrial fibrillation Nelson-Aalen cumulative hazard estimate grouped by dialysis modality. Includes only diagnoses of atrial fibrillation given after dialysis initiation (diagnoses given before were excluded). Groups were defined by the modality of dialysis at day 90 after dialysis initiation (univariate analysis). HD – haemodialysis, PD – peritoneal dialysis, AF – atrial fibrillation.

The incidence of new-onset AF after dialysis initiation was 4.4 per 100 person-years (4.35 per 100 person-years for HD patients and 5.17 per 100 person-years for PD patients). Univariate analysis showed similar rates of new-onset AF in both dialysis modalities (15.2% in the HD group, 12.8% in the PD group, p = 0.27, Fig. 4).

### Predictors for AF-onset – multivariate analysis

Multivariate analysis showed that only two factors were significantly associated with AF-onset in the entire dialysis cohort: older age and enlarged left atrium at dialysis initiation (Table 4). Other echocardiographic parameters and the type of dialysis modality were not significantly associated with AF.

**Table 4:** Predictors for new-onset atrial fibrillation – multivariate analysis.

Variable	H.R.	95% C.I.		P-value
		Lower	Upper	
Age	1.04	1.03	1.06	**<0.001**
Female sex*	1.05	0.75	1.46	0.79
Peritoneal dialysis^†^	0.94	0.88	1.01	0.08
Enlarged left atrium	2.82	2.00	3.99	**<0.001**
Systolic function	1.01	0.99	1.03	0.22
Mitral regurgitation – moderate or severe	1.54	0.97	2.44	0.07

All the factors, including dialysis modality, were evaluated as time varying covariates, with a potential change in each year. Includes only diagnoses of atrial fibrillation given after dialysis initiation (diagnoses given before were excluded). CI indicates confidence interval; and HR, hazard ratio.

^*^Comparing to male.

^†^Comparing to haemodialysis.

### Warfarin treatment in HD patients

Only 23.3% of the HD patients who were diagnosed with atrial fibrillation received warfarin treatment (Table 5). Warfarin use was associated with a higher rate of cerebrovascular events in HD patients with AF, but not with more bleeding.

**Table 5:** Warfarin use in HD patients with AF

Group	No warfarin use after HD initiation (n = 217)	Warfarin use after HD initiation (n = 66)	P-value
Cerebrovascular event (n, %)	28 (12.9%)	16 (24.2%)	**0.026**
Major Bleeding (n, %)	20 (9.2%)	11 (16.7%)	0.090
Intra-cranial haemorrhage (n, %)	3 (4.5%)	7 (3.2%)	0.611

The Group was defined by the diagnosis of atrial fibrillation ever, before or after dialysis initiation. Warfarin usage was defined by purchase of the drug after the initiation of haemodialysis. Cerebrovascular event is defined by ischemic stroke or transients ischemic attack. Major bleeding is defined in the Methods section. All the events occurred after initiation of dialysis. HD–Haemodialysis, AF–atrial fibrillation.

## Discussion

We present here a long-term cohort study describing the burden of AF, its clinical correlates and associations of AF in HD vs. PD. We first stratified the patients by a diagnosis of AF and then by their dialysis modality. To our knowledge, only one study has directly compared the risk for developing new AF between dialysis modalities^4^. In the current study, we investigated chronic dialysis patients, both HD and PD, irrespective of AF presentation. Our methodology was designed to address a switch from one dialysis modality to another during the study.

### Echocardiographic assessment of AF patients

The association between echocardiographic parameters and AF in our study is consistent with the results of previous studies^13,14^. Only enlarged left atrium at the initiation of dialysis, either when adjusted or unadjusted for systolic function, was significantly associated with the development of AF. As noted by Hensen *et al*.^14^, the enlargement of the left atrium reflects the remodelling process of the atrium, which is known to be a risk factor for AF.

### AF associated outcomes

The impact of AF on ischemic stroke incidence in dialysis patients is controversial, in contrast to the general population. Findlay *et al*.^9^ did not find a significant association between AF and stroke in HD patients, but Wizemann *et al*.^15^, using data from the DOPPS (Dialysis Outcomes and Practice Patterns Study), found that pre-existing AF is a significant risk factor for stroke in HD patients. Our results are consistent with those of Findlay *et al*.^9^, although both previous studies did not include PD patients. In contrast to stroke, both univariate and multivariate analyses show that in our cohort AF is independently associated with higher rates of all-cause mortality, consistent with the result of Findlay *et al*.^9^. Thus, it seems plausible that AF-related mortality in dialysis patients is independent of cerebrovascular events but rather may reflect the underlying cardiovascular morbidity. Of note, while the specific cause of death was not reviewed in our study, AF was associated with a higher rate of heart failure, which was identified as a significant predictor of mortality (data not shown).

### Atrial fibrillation and dialysis modality

This study did not find a significant difference in AF incidence between dialysis modalities. Abbott *et al*.^4^ directly compared the incidence of AF in a specific population of HD vs. PD patients, i.e. patients hospitalized with AF. They found that HD patients were at increased risk for developing AF. Shen *et al*.^5^ compared HD and PD patients to patients without ESRD and found a slightly higher rate of AF in the HD group than in the PD group, yet no direct comparison was reported between HD and PD.

Buiten MS *et al*.^16^ and Krueger *et al*.^17^ discussed the main hypotheses for AF development in HD patients involving either volume overload or electrolyte shifts during HD treatment. They showed that HD treatment increases AF occurrence in the short term, but the number of PD patients in these studies was either very small or they were not included. As Goldstein *et al*.^18^ and Korantzopoulos *et al*.^19^ suggested, there are numerous factors that may contribute to AF development in dialysis patients, including volume and pressure overload, chronic inflammation and oxidative stress, activation of the autonomic nervous system and the HD treatment itself. In our cohort, HD and PD patients had similar baseline characteristics at dialysis initiation, in terms of age, sex, echocardiographic parameters and most of the comorbidities. The rate of new-onset AF was also similar between the modalities. Thus, it is possible that the higher risk for AF may be accounted for by comorbidities, such as heart failure and enlarged left atrium, or by the uremic milieu. Further multicentre studies are needed to compare the dialysis modalities and better understand the mechanisms of AF development in dialysis patients.

### Warfarin use for AF in dialysis patients

Warfarin usage in dialysis patients with AF is controversial. Olesen *et al*.^20^ found that warfarin treatment was associated with a decreased risk of stroke or systemic thromboembolism among patients requiring renal-replacement therapy. However, a recent meta-analysis did not find a protective effect of warfarin for stroke prevention in dialysis patients with AF, nor did it find an increased risk for bleeding^21^. In this context, our results of the higher rate of cerebrovascular events in warfarin users may be accounted for by two factors, either an indication bias or the small number of events in both groups. Of note, we could not measure adherence to warfarin treatment, thereby we analysed it as intention-to-treat using prescription purchase data. It should be stated that different meta-analyses found an increased risk of bleeding in these patients due to warfarin use^22,23^. Further studies, specifically randomized controlled trials, are necessary to elucidate the role of warfarin use in dialysis patients with AF.

### Strengths and limitations of the study

One strength of this study has been the retrospective data collection for 14 years from a population cohort, enabling follow-up for most of the patients until their death. In our multivariate analysis the covariates, including the dialysis modality and all comorbidities, are changing in time. Thus, in this analysis every patient was analysed by the dialysis modality and individual comorbidities in each year, thereby designed to overcome confounding of the univariate intention-to-treat analysis. It is worth noting that according to the univariate model, 60% of the patients originally defined in the PD group were eventually treated with HD at least once (average vintage 2.33 ± 2.54 years, Supplementary information Table).

Our study has several limitations. This is a retrospective study conducted in a single centre. Data on patients insured by HMOs other than “Clalit” was not fully available and necessitated exclusion of these patients. The size of the PD cohort was small; therefore, we can’t exclude the possibility of type II error. Some of the data, including echocardiography test results and some laboratory tests were not available for all patients on the same time point. All the diagnoses were based on ICD-9 codes, which do not differentiate between different types of atrial fibrillation (valvular vs. non-valvular disease and chronic vs. paroxysmal) and pose a risk for coding bias.

In conclusion, our study did not find an association between dialysis modality and the development of AF. It may be possible that other comorbidities and the uremic milieu itself may account for the high rates of AF in dialysis patients, and further research is needed to elucidate these mechanisms. The results are clinically relevant since they may help physicians understand that while dialysis patients with AF are at increased risk, the type of dialysis modality does not appear to affect the rate of new-onset AF.

## Electronic supplementary material

Supplementary Dataset 1
