# Supplementary material for: Atrial Fibrillation Characteristics in Patients on Haemodialysis vs. Peritoneal Dialysis
Source: Sci Rep. 2018 Feb 14;8:2976. doi: 10.1038/s41598-018-21229-9 (PMC5813026; doi:10.1038/s41598-018-21229-9)
Supplement: Supplementary file 1 — Supplementary Dataset 1 [file 41598_2018_21229_MOESM1_ESM.pdf]

Scientific Reports, January 2018
DOI: 10.1038/s41598-018-21229-9

Atrial Fibrillation Characteristics in Patients on Haemodialysis vs. Peritoneal Dialysis – Supplementary Information

Ran Abuhasira B.Med.Sc, Yuval Mizrakli B.Med.Sc, Avi Shimony MD, Victor Novack MD PhD, Alla Shnaider MD, Yosef S. Haviv MD

Table

Table legend – Switching between dialysis modalities and dialysis vintage.

**Group**	**All**<br>N = 1,130	**HD group**<br>n = 1,021	**PD group**<br>n = 109	**P-value**
**HD**	1,087 (96.2%)	-	66 (60.6%)	-
**HD vintage** (years)	3.53 ± 2.96	3.60 ± 2.97	2.33 ± 2.54	**.001**
**PD**	169 (15.0%)	60 (5.9%)	-	-
**PD vintage** (years)	1.97 ± 2.40	.66 ± 1.39	2.68 ± 2.53	**<.001**

Groups were defined by the modality of dialysis at day 90 after dialysis initiation. HD – haemodialysis, PD – peritoneal dialysis.
